# Protective Effects of 5-HT1A Receptor Inhibition and 5-HT2A Receptor Stimulation Against Streptozotocin-Induced Apoptosis in the Hippocampus

**DOI:** 10.21315/mjms2019.26.2.5

**Published:** 2019-04-30

**Authors:** Siamak Shahidi, Nasrin Hashemi-Firouzi, Simin Afshar, Sara Soleimani Asl, Alireza Komaki

**Affiliations:** 1 Neurophysiology Research Center, Hamadan University of Medical Sciences, Hamadan, Iran; 2 Department of Biology, Science and Research Branch, Islamic Azad University, Tehran, Iran; 3 Anatomy Department, School of Medicine, Hamadan University of Medical Sciences, Hamadan, Iran

**Keywords:** streptozotocin, apoptosis, hippocampus, 5-HT1A receptor, 5-HT2A receptor, rat

## Abstract

**Introduction:**

Intracerebroventricular administration of streptozotocin (icv-STZ) induced apoptosis changes in neurons similar to Alzheimer’s disease. The serotonergic system via its receptor involved in survival of neurons. The present study examined the ability of selective 5-HT1A receptor antagonist (NAD-299) and 5-HT2A receptor agonist (TCB-2) to attenuate the apoptosis caused by the icv-STZ in the rat.

**Methods:**

The icv-STZ (3 mg/kg, 10 μL, twice) induced neuronal loss in the hippocampus of adult male rats. Animals were divided into naive control, sham-operated, STZ+saline (1 μL, icv), STZ+NAD-299 (5 μg/μL, icv), STZ+TCB-2 (5 μg/μL, icv), and STZ+NAD-299+TCB-2 (5 μg/μL of any agent, icv) groups. Following the 35 days’ treatment period, neuronal apoptosis was detected using the Tunnel. Cells with morphological features of apoptotic cell were contended by microscopy.

**Results:**

TCB-2 and NAD-299 administration decreased number of apoptotic neurons in the treatment group compared with the STZ group. Combined treatment of STZ rat with NAD+TCB more decreased number of apoptotic cells in compare to TCB-2 or NAD-299 treated STZ groups.

**Conclusion:**

Treatment with 5-HT1A receptor antagonist or 5-HT2A receptor agonist diminished apoptosis. The beneficial effect of 5HT1A receptor inhibition was potentiated with activation of 5-HT2A receptor in prevention of apoptosis in hippocampus.

## Introduction

Alzheimer’s disease (AD) is a neurodegenerative disorder (1) that leads to progressive cognitive dysfunction (2–4) and affects different areas of the brain such as the amygdala, entorhinal cortex, and hippocampus (3, 5). The hippocampus, a brain area critical for learning and memory, is a vulnerable and plastic brain structure that is damaged at early stages of AD (6–8). The neuropathology of AD is characterised by extracellular deposits of β-amyloid (Aβ) plaques within senile plaques, intracellular neurofibrillary tangles of tau protein (NFTs) and neurodegeneration (9–11). Accumulation of Aβ plaques in the hippocampus lead to synaptic degeneration (12–14), neuronal apoptosis (15) and cognitive impairment (16, 17).

Intracerebroventricular administration of streptozotocin (icv-STZ) is one of the animal model of AD (18–20). The local injection of STZ, a glucosamine derivative of nitrosourea, at a sub-diabetogenic dose makes similar pathology to AD such as aggregation of Aβ peptides (21, 22), tau hyperphosphorylation, impairment of brain glucose transporters of neurons (23) and increased neuronal death rate (20, 24–26). Apoptosis is a process of programmed cell death (21) and is a basic physiologic process contributing to the maintenance of cellular homeostasis (14, 19, 27, 28). Apoptosis is characterised by cytoplasmic membrane blebbing, cell shrinkage, chromatin condensation and nuclear DNA fragmentation (21, 29). A large number of apoptotic cells can be found in AD tissue (30). Mutations in AD causative genes such as amyloid precursor protein (APP), Presenilin-1 (PSEN1) and Presenilin-2 (PSEN2), increase Aβ peptide (31). In AD, there are several stimuli for apoptosis including reactive oxygen species, accumulation of Aβ (30), mitochondrial dysfunction and DNA damage (31).

Several neurotransmitter systems affected by AD including the acetyl choline, gamma-amino butyric acid (GABA), serotonin and norepinephrine (2, 3, 32). Serotonin (5-hydroxytryptamine; 5-HT) is a multifunctional bioamine acting as a neurotransmitter and a neuromodulator with a particular presence in the hippocampus (33, 34). 5-HT has multiple functions in the mammalian central nervous system such as anxiety, memory, nociception, reward and addiction (35–37). These effects are mediated through seven types of 5-HTR (38). Seven families of 5-hydroxytryptamine receptors and subtypes (5-HT1A–1E, 5-HT2A–2c, 5-HT3A–3C, 5-HT4, 5-HT6, and 5-HT7) have been identified (39). All 5-HTR are G-protein-coupled receptors (GPCRs) with the exception of 5-HT3, which is a ligand-gated cation channel (40).

The highest density of the 5-HT1AR was found in areas important for learning and memory, such as the frontal cortex, hippocampus and septum (41). The 5-HT1AR is to stimulate cell proliferation, differentiation and apoptosis (42). It has been reported that the 5-HT1AR induces apoptosis in CHO cells (43). Several studies show that stimulation of postsynaptic 5-HT1AR in the dorsal raphe counteracts deficit in learning in naive rats (44), while 5-HT1AR antagonists can enhance cholinergic and/ or glutamatergic transmission and improve cognitive functions in various animal models of cognitive dysfunction (45). Over-expression of 5-HT1AR on a rat model of AD was demonstrated (46).

The 5-HT2A receptors are remarkably expressed in the hippocampus (33). It has been reported that 5-HT2AR agonists improved learning and memory impairments, while 5-HT2AR antagonists have anti-psychotic and anti-depressant properties (47). Recently, it was found that 5-HT1AR blockade and 5-HT2AR activation improved cognitive dysfunction in icv- STZ-treated rats (48). However, data regarding 5-HT1AR inhibition and 5-HT2AR activation in programme cell dead in AD rat is not available.

The previous study used Nissl staining in order to quantify the neuronal loss (48). The Nissl staining show nucleic acid content of cells (49). Neurons have DNA in the nuclei and RNA highly RNA concentrated in rough endoplasmic reticulum and ribosomes (Nissl substance) (50) that stained with cresyl violet in this method (51). Due to Nissl staining technique is based on the binding of basic dye with the nucleic acid content of cells (50), it is not a specially distinguished nuclei of dead neuron (52). Terminal Deoxy nucleotidyl Transferase-Mediated dUTP Nick-End Labeling (TUNEL) staining is a suitable method for detecting DNA fragments of nuclei (53). Therefore, Tunnel is very useful method to study the nuclei of neurons, and understand the apoptotic neurons of the brain area. The aim of this study was to determine the chronic effect of 5-HT1AR antagonist and 5-HT2AR agonist, on the rate of apoptosis in the hippocampus area in a rat model of AD.

## Materials and Methods

### Animals

Adult male Wistar rats (250 g–300 g) were obtained from the animal house of Hamadan University of Medical Sciences. All animals were housed in a room with temperatures ranging from 20 °C–24 °C and lights maintained on a 12:12 light: dark cycle. Rats were allowed to acclimate for one week prior to the study. Water and food were available ad libitum. All experiments were approved by the research and ethics committees of the Hamadan University of Medical Sciences (IR.UMSHA.REC.1395.547) and were performed according to the Guide for Care and Use of Laboratory Animals published by the United States National Institutes of Health (NIH Publication No. 85-23, revised 1985).

### Chemicals

STZ was purchased from Santacruz Company (CA, USA). NAD-299, [(R)-3-N, N-dicyclobutylamino-8-fluoro-3,4-dihydro-2H-1-benzopyran-5-carboxamide hydrogen (2R,3R)- tartrate monohydrate; AZD7371] and TCB-2 [(7R)-3-bromo-2,5-dimethoxy-bicyclo[4.2.0] octa-1,3,5-trien-7-yl] methanamine] were purchased from Tocris Bioscience Company (Bristol, UK). NAD-299 and TCB-2 were dissolved in dimethyl sulfoxide (DMSO) and STZ was dissolved in normal saline.

### Study Design

**Figure 1:**
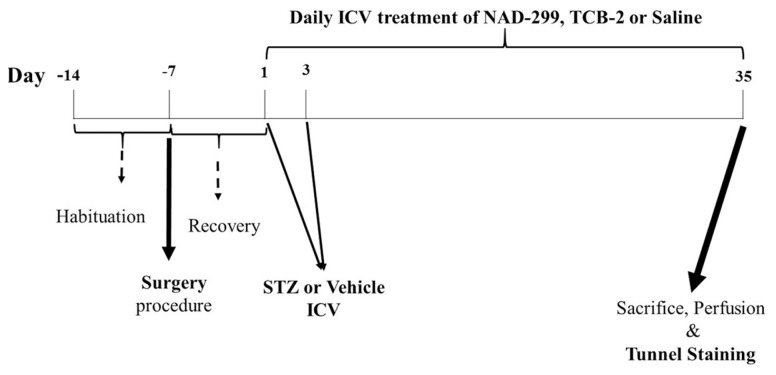
Time line of experimental design

The rats were divided randomly into the following six groups (*n* = 5 rats in each group): (i) control group, which did not undergo operation or treatment; (ii) sham group, which received 10 μL of vehicle via icv injection during operation and subsequently received 1 μL of vehicle for treatment; (iii) AD group, which received STZ (3 mg/ kg, 10 μL) via intracerebroventicular (icv) injection during operation and subsequently received 1 μL of vehicle treatment for 35 days; (iv) AD+NAD-299-299 group, which received STZ via icv injection during operation and subsequently received NAD-299 (selective 5-HT1A R antagonist, 5 μg/μL); v) AD+TCB-2 group, which received STZ via icv injection during operation and subsequently received TCB-2 (selective 5-HT2AR agonist, 5 μg/μL) and vi) AD+TCB-2+NAD-299 group, which received STZ via icv injection during operation and subsequently received TCB-2 (5 μg/0.5 μL) and NAD-299 (5 μg/0.5 μL). All of treatments, (TCB-2, NAD-299 and vehicle) were done via icv injection for 35 days. Figure 1 shows a schematic of experimental design and the timeline.

### Surgical Procedures

The animals were anesthetised with ketamine (100 mg/kg, Behbod Darou, Iran) and xylazine (10 mg/kg, Alfasan, The Netherlands) and placed in a stereotaxic apparatus (Stoelting Co., Chicago, IL). The head positioned in a frame and a midline sagittal incision was made in the scalp. A guide cannula was lowered into the right lateral ventricle using the following coordinates: −0.9 mm posterior to the bregma, 1.6 mm lateral to the sagittal suture, and 3.1 mm beneath the skull (54). The guide cannula was secured for icv injection. After surgery procedure, the rats were recovery for one week. Seven days’ recovery prevented inflammatory reaction in rats. For an overview of the experimental protocol and timeline, see Figure 1.

**Figure 2:**
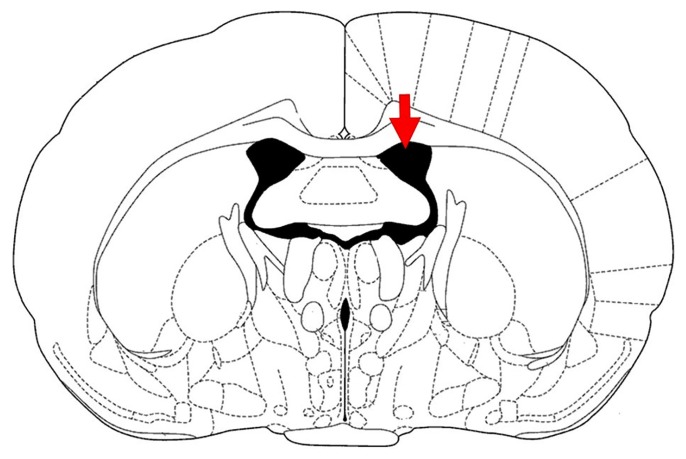
Illustration of rat brain section. The approximate location of STZ, NAD-299, TCB-2 or saline icv injection in a cross section view of atlas plate

To create AD model, STZ was dissolved in 0.9% saline, then immediately divided into aliquots and stored at −20 °C before use. STZ microinjected icv after 7 days of recovery (day 1) and again 2 days later via a cannula (day 3) at a dose of 3 mg/kg in 10 μL (34, 55, 56). After STZ microinjection, animals were icv treated NAD-299, TCB-2 or vehicle for 35 consecutive days. Drugs or vehicle microinjections were performed with a 30-G injector cannula (1 mm below the tip of the guide cannula) with a Hamilton syringe (Hamilton, Bonaduz, Switzerland) attached to the injector cannula by polyethylene micro-tubing (PE-20). Figure 2 shows the positioning of the treatment cannulas and the STZ, NAD-299, TCB-2 and saline injection.

### Tissue Preparation

At the end of treatment, all the animals were anesthetised with ketamine and xylazine (100:10 mg/kg), transcardially perfused with 4% paraformaldehyde in 0.1 M phosphate buffer (pH 7.3) and their brains were dissected out (Figure 1). Isolated rat brain was fixed into 4% paraformaldehyde for 1 week and brains were dipped into paraffin. The brain sliced into 5 μm tissue sections using a microtome (LeitzGmBH, Wetzlar, Germany). The hippocampi were sectioned into 5 μm with 120 μm intervals (57).

### TUNEL Staining: Determination of Neuronal Apoptosis

To identify apoptotic cell death in the hippocampal neurons, TUNEL staining was performed using the kit (Roch, Germany) according to the manufacturer’s instructions. The average apoptotic cell number was prepared for DNA fragmentation detection using the assay, as previously described (58). According to Paxinos and Watson (54) the coordinates for analysing the CA1 hippocampal region are −3.3 to −3.8 from Bregma, similar as previously described study (59). In brief, after sample permeabilisation (0.1 M citrate buffer, pH 6), sections were incubated with TUNEL reaction mixture for 60 min at 37 °C. Following this, converter-peroxidase (30 min) and 3,3’-diaminobenzidine substrate (10 min) were added to the samples, in that order. Sections were counterstained for hematoxylin. Mounted sections were photographed with a digital camera attached to a light microscope (400×) and number of the brown dark cells was counted. For each animal, the mean apoptotic cell number was obtained by counting five coronal sections.

### Statistical Analysis

The data were analysed with one-way analysis of variance (ANOVA) and Tukey post-hoc tests. Statistical significance was set at *P* < 0.05. The data are expressed as mean ± standard deviation.

## Results

**Figure 3:**
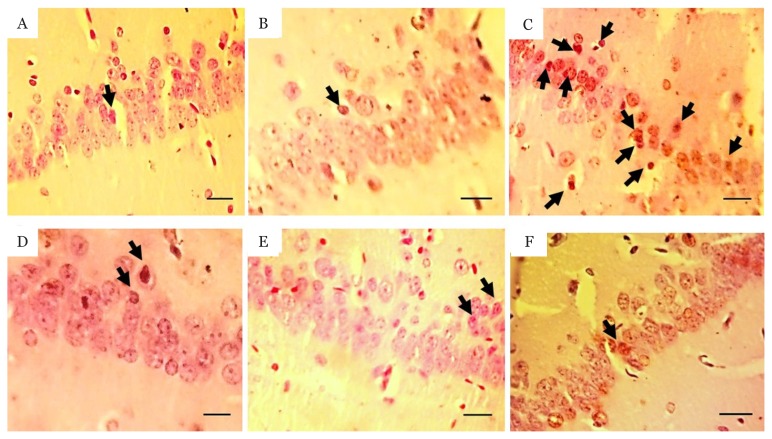
Light micrographs of cell apoptosis in the hippocampal CA1 region. Sections derived from (A): control; (B): sham; (C): STZ; (D): STZ+TCB-2; (E): STZ+NAD-299 and (F): STZ+TCB+NAD groups stained by TUNEL. The arrow shows the apoptotic neuron. Scale bar = 100 μm, magnification: 400×

**Figure 4:**
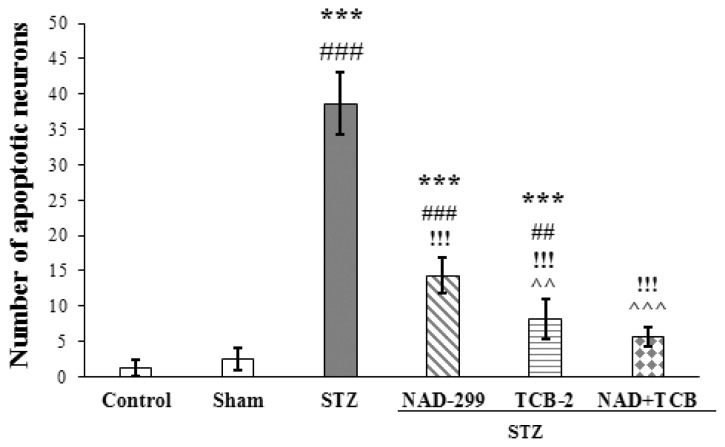
The number of apoptotic neurons was calculated. Each column represents mean ± SDM. *** (*P* < 0.001) as compared with control group. ###, (*P* < 0.001) and ##, (*P* < 0.01) as compared with sham group. !!!, (*P* < 0.001), as compared with STZ group. ^^^, (*P* < 0.001) and ^^, (*P* < 0.01) as compared with STZ+NAD group

Immunohistochemistry for apoptotic neurons in the CA1 region of the hippocampal coronal sections was showing in Figure 3 (a–f). TUNEL staining was further performed in these sections to detect apoptotic neurons. Table 1 presented number of apoptotic neurons for CA1 area of the hippocampus in all groups. One-way ANOVA has detected a significant difference in the number of apoptotic cells in the CA1 region between experimental groups [F(5,35) = 174.06, *P* < 0.001] (Figure 4). Tukey post-hoc analysis revealed a higher number of apoptotic neurons in the STZ group, than in the control, sham and STZ treated with NAD- 299, TCB-2 and NAD+TCB groups, respectively (*P* < 0.001). These results showed a significant reduction in apoptotic neurons in the STZ group treated with NAD+TCB and STZ rats treated with TCB-2 when compared with the NAD-299 group (*P* < 0.001 and *P* < 0.003, respectively). Also, the number of apoptotic cells was significantly lower in the STZ group treated with NAD+TCB than in the untreated STZ group (*P* < 0.001). Tukey post-hoc analysis showed that number of apoptotic neurons increased in the STZ treated with NAD-299 and TCB-2 groups compare to sham group (*P* < 0.001; *P* < 0.003, respectively). The apoptotic cell numbers were not significantly different between control and sham groups (*P* = 0.969 < 0.050).

## Discussion

The present study evaluated the effects of 5-HT1AR inhibition and 5-HT2AR activation by selective antagonist and agonist in a rat model of AD. The result of current study showed that neuronal apoptosis induced by icv injection of STZ in the hippocampus of AD rat. Treatment with NAD-299 (selective, high affinity 5-HT1AR antagonist), TCB-2 (potent, high affinity 5-HT2AR agonist), and NAD-299+TCB-2 in rats receiving STZ, decreased the neuronal apoptosis in the hippocampus area.

Hippocampus has a critical role on the learning and memory (60). Synaptic plasticity in the hippocampal neurons involves in the memory formation (4). Several agents as well as neurotransmitters such cholinergic, glutamatergic, serotonergic, GABAerigic, vanilloid, cannabinoid systems influence the hippocampal synaptic plasticity and learning and memory (3, 60, 61). In the AD, memory impairments are due to the hippocampal neurodegeneration, imbalance of neurotransmitter systems and apoptosis in neurons (14, 62, 63).

Present data confirmed that twice administration of STZ into the brain induces loss of neurons in the hippocampus (64), and the neuron death done (14, 28). In a recent study, the results of Nissl staining showed neuronal death in the hippocampus of STZ treated rats (48) while it was not recognised the necrosis and apoptosis. TUNEL staining is a valuable technique to recognition of apoptotic neuron in the brain areas such as hippocampus (55, 65, 66). TUNEL staining confirmed that apoptosis occurred after icv-STZ administration in rat brain (67, 68). DNA fragmentation exhibited the apoptosis condition in the brain (29, 69). Icv injection of STZ caused oxidative stress and neuronal apoptosis in rat brain by proapoptotic pathway (69, 70, 71). In addition, icv STZ initiated inflammation mechanisms in the cell (72, 73). However, there is no neuroinflammation data in this study which to be construed details.

The present study revealed that icv injection of NAD-299 diminished the effect of STZ-induced neuronal apoptosis in the hippocampus. 5-HT1AR protein expressions decreased neuronal death of astrocytes in the ischemic hippocampal CA1 region (74). In agreement with current results, amyloid beta peptide induced neuronal death and decreased expression of 5-HT1AR in rat brain (46). Another study indicated that 5-HT1A receptor stimulates both of anti-apoptotic and pro-apoptotic pathways in hamster ovary fibroblast cells (43). Stimulation of 5-HT1AR in the dorsal raphe counteracts the effect of intrahippocampal 7-chloro-kynurenic acid micro-injection on pyramidal cells in the hippocampus (44). Upregulation of 5-HT1AR by the non-selective 5-HT1AR agonist, 8-OH-DPAT alleviate cellular apoptosis, and downregulation of 5-HT1AR mediated the apoptosis pathway in the hippocampus of mouse brain (75).

The 5-HT1AR is highly expressed in the hippocampus (2, 33). This receptor is related to inhibitory Gi-proteins and 5-HT1AR function result to an inhibition of adenylyl cyclase’s activity and thus decrease the cAMP production (76). Binding of antagonists to 5-HT1AR cause disinhibitory effect of 5-HT (77, 78), and increase the activity of serotonergic neurons (79). Hippocampal 5-HT1AR expression changed in AD, and molecular binding to 5-HT1AR has been examined in AD patients (46). Present finding showed that NAD-299, selective 5-HT1AR antagonist decreased the neuronal apoptosis in hippocampus. Also, this study confirmed the previous suggestion about selective agonist for 5-HT1AR and new therapeutic target in nervous disorders (39) and it is demonstrated that Aβ injected in rat hippocampus induced over expression of 5-HT1AR a rat model of AD (46).

The other finding of current study showed that icv administration of TCB-2, potent and high affinity 5-HT2AR agonist, reduced STZ-induced apoptosis in the hippocampus. 5-HT2AR has high expression in the hippocampus (33). The expression of 5-HT2AR mRNA in granule cells and pyramidal neurons of hippocampus have been demonstrated in clinical and pre-clinical cellular studies (80, 81). TCB-2 binding to the 5-HT2AR is linked to Gq-proteins and exert its effect via the phospholipase-C signaling pathway (74), and 5-HT2AR activity prevents apoptosis of cell (46). Therefore, it seems that, chronic icv administration of TCB-2 decreased the apoptosis in neurons of hippocampus.

Current result indicated that treatment with NAD-299+TCB-2 inhibited the apoptosis effect of icv-STZ in the hippocampus of rat. It seems that blockade of 5-HT1AR and the activation of 5-HT2AR can potentiate the valuable, and single effect of NAD-299 or TCB-2 in neurons of hippocampus. There are few available studies about synergetic effect of these receptors and apoptosis in the nervous system. Both of these receptors have the remarkable expression of mRNA in the hippocampus (81). Combined treatment with NAD-299 and TCB-2 begin phospholipase-C signaling pathway, and reinforce the influence of serotonin in serotonergic neurons (77–79). The technique or agent which can modulate the death of a cell is known for therapeutic target in neurodegenerative disease (21). Therefore, current finding showed that the combined treatment with NAD-299 and TCB-2 has a synergic effect in the brain and the apoptosis neuron is decreased in the hippocampus of rats.

There are some limitations in the current study. One of the limitations is other assays support the results of TUNEL assay in order to differentiate the cell necrosis from neuronal apoptosis in this study. Also, the result of current study is completed, if the control with NAD-299 and TCB-2 treated without icv-STZ injection is added in experimental groups. The other limitation of this finding was the treatment doses of TCB-2 and NAD-299. There was no significant difference in STZ+TCB-2 compared to STZ+NAD+TCB. Synergistic effect of 5-HT1AR inhibition and 5-HT2AR stimulation was found compared to 5-HT1AR blocking. It is possible that findings were completed by different doses of agent. The more research is necessary for better understanding about the beneficial effect of synergic treatment of these receptors in the central nervous system.

## Conclusion

In conclusion, the present study demonstrates that 35 days icv administration of NAD-299 and TCB-2 prevented the STZ-induced apoptosis in the hippocampus of rat received icv-STZ. The combine treatment with NAD-299 and TCB-2 has a synergic effect in the hippocampus.

## Figures and Tables

**Table 1:** Number of apoptotic neurons for CA1 area of the hippocampus

Group	Control	Sham	STZ	STZ+NAD	STZ+TCB	STZ+NAD+TCB
Mean	253.16	218.5	83.3	132.66	153.83	148.83
SEM	6.46	15.39	6.18	7.03	5.85	6.18
SDM	15.82	37.708	15.13	17.23	14.34	15.14

standard error mean (SEM), standard deviation mean (SDM)
